# How can motor systems retain performance over a wide temperature range? Lessons from the crustacean stomatogastric nervous system

**DOI:** 10.1007/s00359-014-0975-2

**Published:** 2015-01-01

**Authors:** Eve Marder, Sara A. Haddad, Marie L. Goeritz, Philipp Rosenbaum, Tilman Kispersky

**Affiliations:** Volen Center and Biology Department, MS 013, Brandeis University, 415 South St., Waltham, MA 02454 USA

**Keywords:** Crustaceans, *Q*_10_, Acclimation, Stomatogastric ganglion, *Cancer borealis*, Crabs, Neuromodulation, Pyloric rhythm

## Abstract

Marine invertebrates, such as lobsters and crabs, deal with a widely and wildly fluctuating temperature environment. Here, we describe the effects of changing temperature on the motor patterns generated by the stomatogastric nervous system of the crab, *Cancer borealis*. Over a broad range of “permissive” temperatures, the pyloric rhythm increases in frequency but maintains its characteristic phase relationships. Nonetheless, at more extreme high temperatures, the normal triphasic pyloric rhythm breaks down, or “crashes”. We present both experimental and computational approaches to understanding the stability of both single neurons and networks to temperature perturbations, and discuss data that shows that the “crash” temperatures themselves may be environmentally regulated. These approaches provide insight into how the nervous system can be stable to a global perturbation, such as temperature, in spite of the fact that all biological processes are temperature dependent.

## Introduction

Ambient temperature influences all living organisms, but none more than long-lived marine crustaceans. Temperatures in the north Atlantic Ocean, where the lobster, *Homarus americanus* and the crab, *Cancer borealis*, live, routinely fluctuate from below 4 °C to more than 25 °C (Factor 1995; Crossin et al. 1998; Camacho et al. 2006). Moreover, these animals can experience substantial temperature swings in very short times as a consequence of tides, currents, and storms. For those of us who use wild-caught marine crustaceans as experimental systems, it is important to understand the environmental challenges they face, as well as to appreciate that they also show temperature preferences that can contribute to where they choose to live (Krediet and Donahue 2009; Lewis and Ayers 2014).

Temperature is a global perturbation that influences all biological processes, to a greater or lesser degree. Biologists often express the temperature dependence of a specific process by calculating the *Q*_10,_ the factor by which a process increases for every 10 °C increase in temperature. A *Q*_10_ = 1 characterizes a process that is essentially temperature invariant. Many biological processes, including most voltage-gated ion channels, have activation and inactivation rates with *Q*_10_s in the 2–3 range, while the ion channels that are thought important for temperature sensing may have *Q*_10_s as high as 50 or 100 (Garrity et al. 2010; Clapham and Miller 2011; Kang et al. 2012).

Importantly, if all of the component processes that govern a system’s behavior have identical *Q*_10_’s, the process will be temperature compensated (Robertson and Money 2012). For example, if all of the *Q*_10_s that control the activation and inactivation kinetics of the ion channels contributing to action potential generation are the same, then the action potential waveform will be maintained as temperature is changed (although the frequency may change). However, as the *Q*_10_ describes an exponential function, a relatively modest difference in the *Q*_10_s for two processes can nonetheless become quite significant in response to even a modest temperature perturbation. In this case, maintaining constant performance over a considerable temperature range becomes a non-trivial problem (Tang et al. 2010, 2012; Caplan et al. 2014), and there are relatively few combinations of conductance densities and *Q*_10_s that allow a neuron to function over a range of temperatures (Wechselberger et al. 2006; Rinberg et al. 2013; Caplan et al. 2014; Roemschied et al. 2014). At the same time, sensory and motor function often depends on relative temperature invariance (Tang et al. 2010; Roemschied et al. 2014; Soofi et al. 2014), so it becomes a challenge to understand how physiological performance can be preserved despite the fact that all the components of circuits in behavior are altered differentially by temperature.

## The effects of temperature on the crab pyloric rhythm

The effects of acute temperature change on the triphasic pyloric rhythm have been studied both in vivo (Soofi et al. 2014) and in vitro (Tang et al. 2010, 2012). In both cases, the frequency of the pyloric rhythm increases as the temperature increases over a permissive range, while the phase relationships, or the relative timing, among the component neurons are maintained (Tang et al. 2010). In considering motor systems, it is clear that phase maintenance is critical for appropriate movements, which result from muscle groups being activated in the correct order. The problem of maintaining phase despite changes in frequency has attracted a great deal of attention precisely because it requires understanding how biophysical processes which would tend to produce fixed delays can be combined instead to produce fixed phases (Harris-Warrick et al. 1995a, b; Hooper 1997a, b; Manor et al. 2003; Bucher et al. 2005; Marder and Bucher 2007; Goaillard et al. 2009).

The pacemaker kernel of the pyloric rhythm consists of the 2 PD neurons and a single AB neuron, which are tightly electrically coupled and are synchronously active (Marder and Bucher 2007). Rinberg et al. (2013) pharmacologically isolated the pacemaker kernel and then characterized the effects of temperature. As expected, as temperature was increased, the frequency of the pacemaker oscillation increased. Unexpectedly, the duty cycle of the pacemaker kernel remained virtually constant over a large range of temperatures (Rinberg et al. 2013). This result goes a long way to explain the compensation of phase over temperature previously reported (Tang et al. 2010), because the pacemaker kernel will provide rhythmic inhibition whose duration changes as the period changes. The response of the follower LP and PY neurons to rhythmic inhibitory drive depends on the strength and time course of the inhibition they receive (Eisen and Marder 1982; Rabbah and Nadim 2007) as well as the properties of I_A_ and I_H_ in the follower neurons (Hartline and Gassie 1979; Harris-Warrick et al. 1995b). All of these three processes are temperature dependent, with somewhat different *Q*_10_s (Tang et al. 2010). Nonetheless, they balance well enough to maintain a constant phase (Tang et al. 2010).

**Fig. 1:**
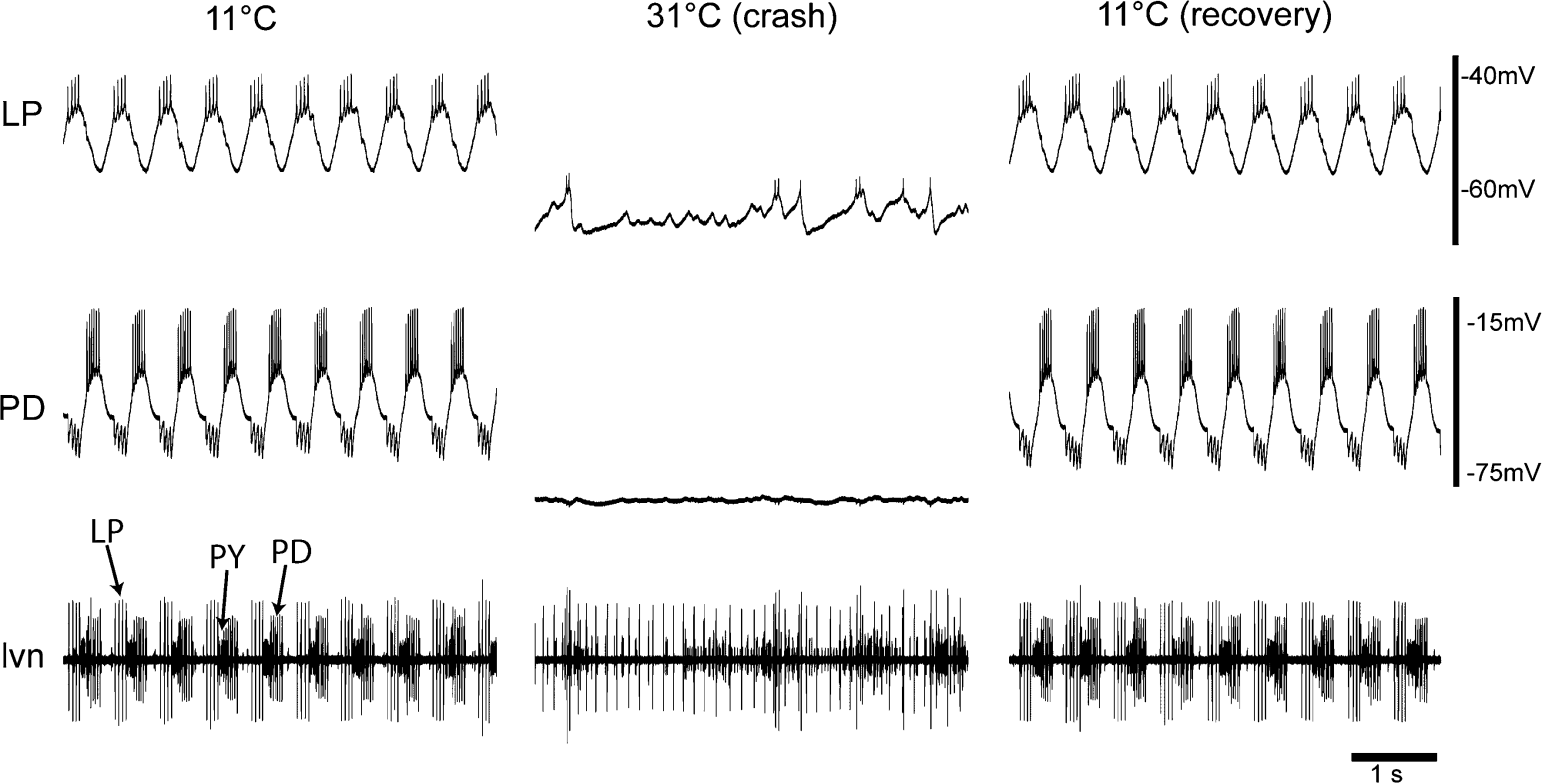
Extreme temperature “crashes” the pyloric rhythm. Example intracellular (*top LP* and* PD*) and extracellular (*bottom lvn*) traces from a preparation at 11 °C (stable rhythm), 31 °C (network crash) and then back to 11 °C (recovery of a stable rhythm)

As the temperature is increased beyond the permissive range, the preparations “crash” or lose their ability to maintain robust triphasic rhythms (Tang et al. 2012). Pyloric rhythm crashes can be reversible (Tang et al. 2012). Figure 1 shows intracellular recordings of the PD and LP neurons and an extracellular recording of the lateral ventricular nerve (lvn, which also shows activity of the PY neurons), first at 11 °C, then at 31 °C, and then back at 11 °C. Note the normal alternation of the LP, PY, and PD neurons at low temperatures, and then the disruption of the rhythm at 31 °C, associated with loss of PD neuron activity, almost complete loss of LP neuron activity, but continued tonic firing in the PY neuron.

Detailed analyses of high-temperature crashes using spectral analyses (Tang et al. 2012) show that individual preparations respond similarly to temperature changes in the permissive range, but crash differently at more extreme temperatures, as was predicted from studies that showed variability in network components (Golowasch et al. 1999; Goldman et al. 2001; Marder and Goaillard 2006; Schulz et al. 2006, 2007; Goaillard et al. 2009; Marder 2011).

## How do isolated neurons crash?

In considering how the pyloric rhythm crashes at high temperatures, we were curious to determine how each component of the pyloric rhythm responds to extreme temperatures. At high temperatures, the isolated pacemaker kernels crashed (Rinberg et al. 2013), but each preparation showed different crash dynamics. Again, the behavior of the isolated pacemaker kernels was robust over a large permissive temperature range, but showed the effect of their underlying differences in conductance parameters as the oscillators made different transitions when they lost their ability to oscillate (Rinberg et al. 2013).

**Fig. 2:**
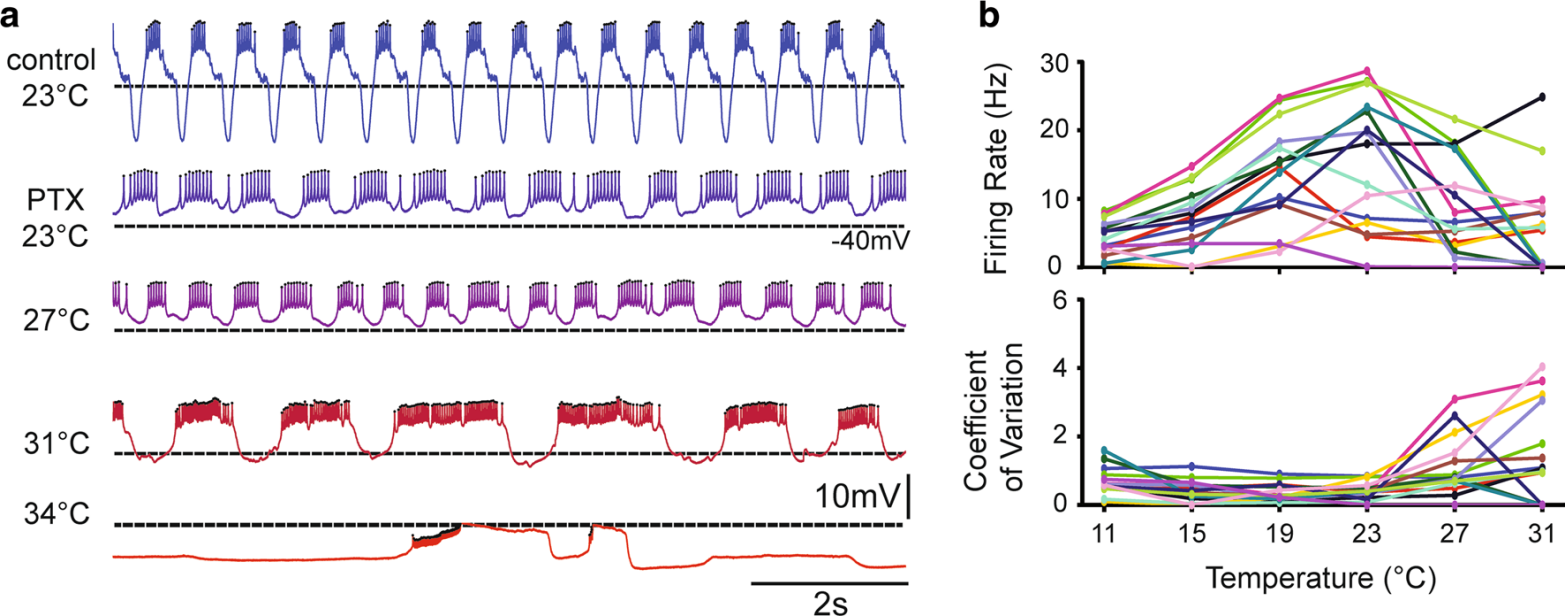
Effect of temperature on the activity of isolated LP neurons. **a** Example traces from one LP neuron. Control, LP is active in the intact network. After applying picrotoxin (PTX), glutamatergic connections to AB and PY are blocked and some residual PD inhibition is left. With increasing temperature, LP spike frequency increases and pauses between LP bursts become larger. At 34 °C LP “crashes”. **b** Firing rate in isolated LP neurons increases with increasing temperature. At 23 °C individual frequencies become more variable due to crashes. The coefficient of variation (ratio between the standard deviation and the mean of the spike frequency) increases at higher temperatures, due to variable spiking patterns in individual LP neurons. *N* = 15 LP neurons; each *color* represents one LP neuron

When the circuit was intact, in many preparations it appeared that the LP neuron was often the first neuron to become silent or highly irregular at high temperature (Tang et al. 2012). Consequently, we decided to pharmacologically isolate the LP neuron and study the effects of temperature on it. The results of one such experiment are shown in Fig. 2a. The top trace, labeled control, shows an intracellular recording of the LP neuron in the intact network. Subsequently, the preparation was placed in picrotoxin to block the glutamatergic inputs to the LP neuron from the AB and PY neurons (Marder and Eisen 1984). The second trace in Fig. 2 shows the absence of large IPSPs in comparison to the control, but that the LP neuron was still firing rhythmically, most likely because of a small inhibitory input remaining from the cholinergic PD neurons (Marder and Eisen 1984). As the temperature was further increased, the LP neuron started generating very long bursts and eventually stopped firing action potentials. Figure 2b shows the pooled data from 15 LP neurons as a function of temperature. Note the individual variation in the effect of temperature on firing rate. Most neurons increased in firing frequency as temperature was raised from 11 to 23 °C, but as the temperature was increased further many of them decreased or stopped firing. The effect of temperature on the coefficient of variation of the firing rate demonstrates clearly that as the temperature reaches 27 °C, the neuronal activity patterns become highly variable, again as would be expected given the variable conductance densities of the LP neurons’ voltage-gated currents (Schulz et al. 2006; Goaillard et al. 2009).

## Temperature acclimation of the pyloric rhythm

There is an enormous literature that demonstrates that animals acclimate to extended periods of time at different temperatures (Lehtikoivunen and Kivivuori 1994; Camacho et al. 2006; Robertson and Money 2012; Tang et al. 2012). We acclimated crabs for 3–4 weeks to low and high temperatures and found little change in the response to acute temperature ramps of in vitro preparations in the permissive temperature range, but found that preparations from warm-acclimated animals required higher temperatures to “crash” the pyloric rhythm (Tang et al. 2012).

**Fig. 3:**
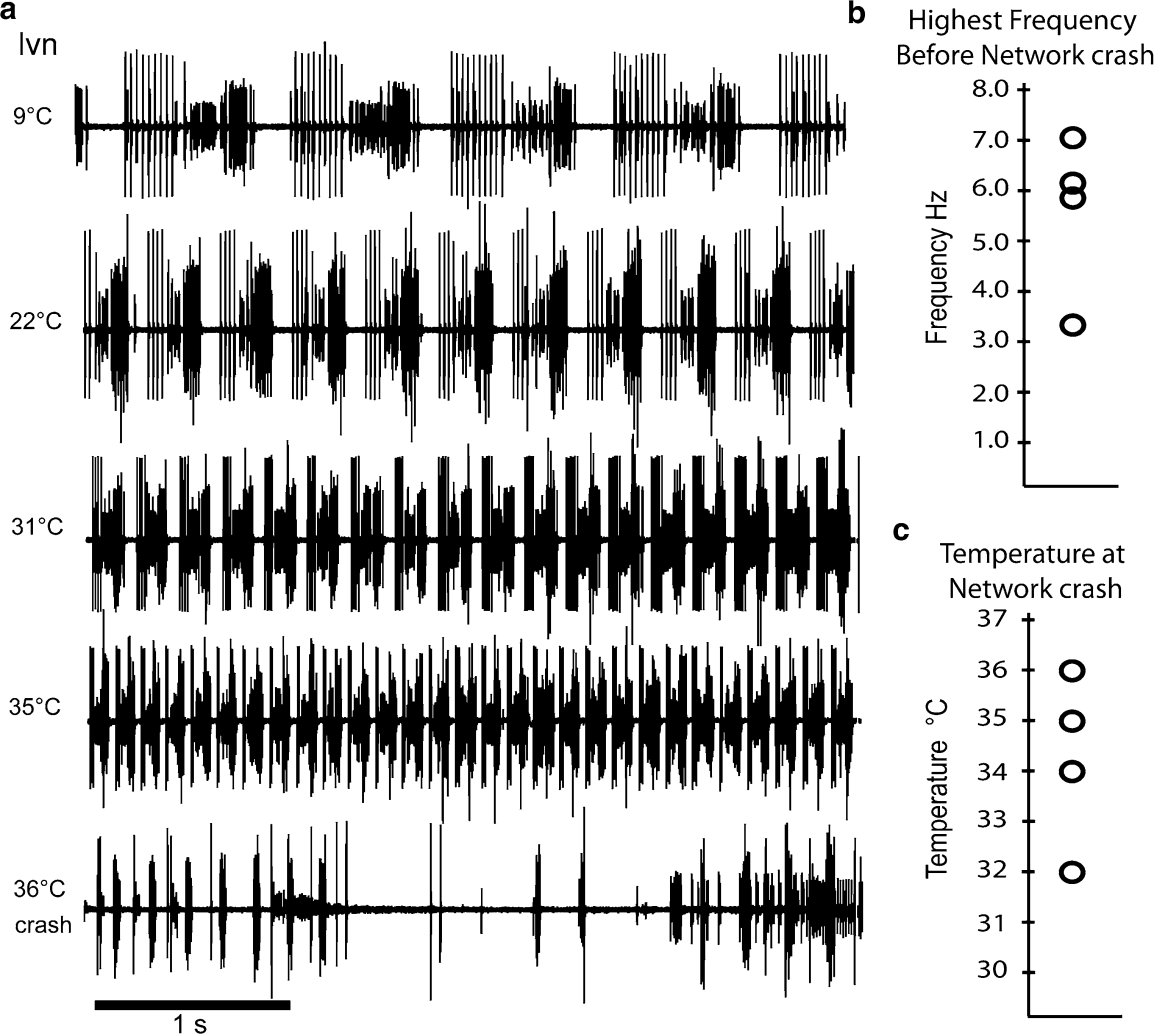
Unusually warm winter ocean temperatures alter the crash point of in vitro preparations. **a** Example extracellular traces from the lvn of a preparation at indicated temperatures. The network reached a pyloric frequency of 7 Hz at 35 °C and then crashed at 36 °C. **b** Cumulative data from four preparations showing the highest recorded frequency before network crash. **c** The temperatures at which the networks crashed from the same preparations

The winter of 2011–2012 was unusually warm and ocean temperatures in the waters from which our animals were collected were 6–8 °C warmer than normal, thus creating a natural acclimation period of many months. Preparations from animals that had lived for extended periods of time in the ocean at much warmer temperatures than usual showed remarkable behavior in response to temperature. Figure 3a shows recordings of the pyloric rhythm in response to acute temperature changes. Note that the frequency of the pyloric rhythm at low temperatures is entirely normal. But, as the temperature reached 31 °C, the frequency was almost 5 Hz, and at 35 °C, it reached almost 7 Hz (Fig. 3a). These frequencies are far greater than we had ever seen previously. Finally, at 36–37 °C, the pyloric rhythm crashed, but at a much higher temperature than we had ever seen in previous years. Data from four preparations from this group of crabs that had over-wintered in warm waters are summarized in Fig. 3b, c, and show the unusually high frequencies and crash points for these animals. Thus, we conclude that an extended period of time at higher than usual winter water temperatures had shifted upwards the temperature tolerance of the animals. (We should note that in the two subsequent years, the typical crash temperatures returned to lower values.)

Crustacean neuromuscular junctions are notoriously temperature sensitive (Stephens and Atwood 1982), with muscle tension and contraction compromised with temperature increase (Hamilton et al. 2007; Thuma et al. 2013). Interestingly, circulating neuromodulators, such as dopamine and serotonin, may play a role in vivo to compensate for the potential loss of function at high temperature (Hamilton et al. 2007; Thuma et al. 2013).

Therefore, we were curious to compare directly the effect of temperature on pyloric rhythms recorded both in vivo with implanted electrodes with results from dissected preparations in vitro (Soofi et al. 2014). At the same temperature, the in vitro recorded motor patterns were faster than what was seen in vivo from the same animal. The steeper temperature dependence of the pyloric rhythm frequency in the in vitro preparations when compared to the in vivo preparations suggests that sensory feedback may reduce the overall effect of high temperature on pyloric rhythm frequency in vivo (Soofi et al. 2014).

**Fig. 4:**
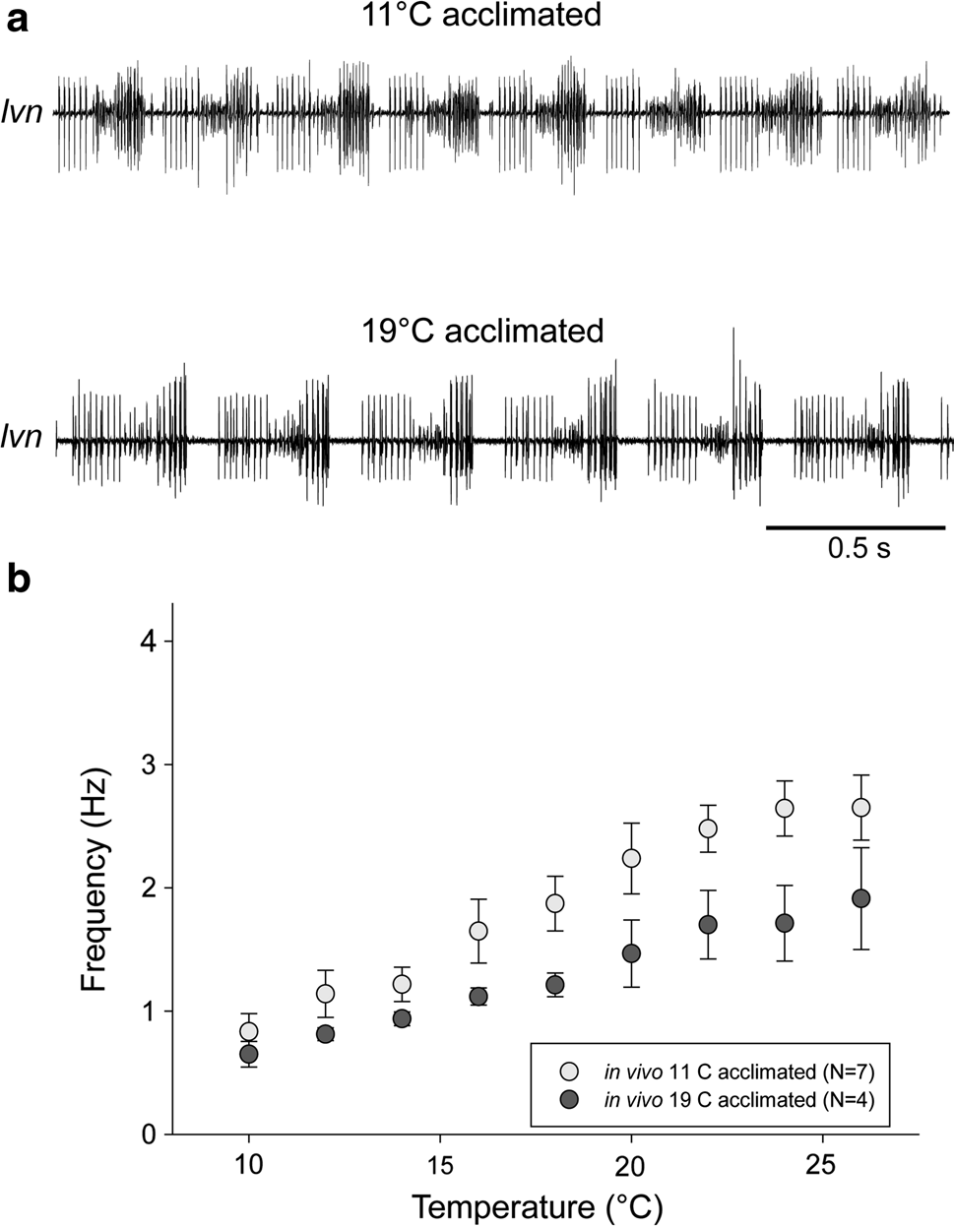
Comparison of pyloric motor patterns recorded in vivo in animals acclimated to different temperatures. **a** Pyloric rhythms recorded at 24 °C in animals that had been acclimated to 11 °C (*top*) and 19 °C (*bottom*), recorded with an implanted extracellular electrode. **b** Pooled and binned data from in vivo recordings in seven cold-acclimated and four warm-acclimated animals. Warm-acclimated animals responded with a significantly shallower increase in frequencies as a function of temperature elevation (two-way ANOVA, *p* < 0.01)

Because the pyloric rhythm frequencies recorded in vivo at high temperature appeared somewhat lower than those produced by the same animals after dissection and in vitro recordings, we were curious to examine the effects of temperature acclimation on the motor patterns recorded in vivo after the animals had been acclimated to higher temperatures for at least 1 month. Figure 4a shows a comparison of the motor pattern recorded at 24 °C from implanted electrodes in animals that had been acclimated for 4 weeks to 11 and 19 °C, respectively. Figure 4b shows that warm-acclimated animals became less sensitive to acute temperature increases in the permissive range. This may be physiologically advantageous, as muscle contraction is more likely to follow the motor pattern discharge when the pyloric rhythm frequency is less than 2 Hz (Morris and Hooper 1997, 1998, 2001), although it would be interesting to see how long-term acclimation changes the properties of the neuromuscular junctions of the pyloric muscles.

## Conclusions

Maintaining a robust pyloric rhythm over a wide temperature range requires that many biophysical, biochemical, and molecular processes have temperature dependencies that collectively preserve the important features of circuit function. While this is difficult to achieve when building computational models (Caplan et al. 2014), there must be many, yet to be discovered, biological rules that allow developing and growing animals to find sets of network parameters that allow them to retain stable circuit function in a temperature-fluctuating ocean.
